# Fibroblast Differentiation and Matrix Remodeling Impaired under Simulated Microgravity in 3D Cell Culture Model

**DOI:** 10.3390/ijms222111911

**Published:** 2021-11-02

**Authors:** Jiranuwat Sapudom, Mei ElGindi, Marc Arnoux, Nizar Drou, Anna Garcia-Sabaté, Jeremy C. M. Teo

**Affiliations:** 1 Laboratory for Immuno Bioengineering Research and Applications, Division of Engineering, New York University Abu Dhabi, Abu Dhabi P.O. Box 129188, United Arab Emirates; jiranuwat.sapudom@nyu.edu (J.S.); me95@nyu.edu (M.E.); anna.sabate@nyu.edu (A.G.-S.); 2 Core Technology Platforms, New York University Abu Dhabi, Abu Dhabi P.O. Box 129188, United Arab Emirates; mga5@nyu.edu; 3 Center for Genomics and Systems Biology, New York University Abu Dhabi, Abu Dhabi P.O. Box 129188, United Arab Emirates; nd48@nyu.edu; 4 Department of Mechanical and Biomedical Engineering, New York University, Brooklyn, NY 11201, USA

**Keywords:** microgravity, 3D cell culture, fibroblast differentiation, tissue repair, matrix remodeling

## Abstract

Exposure to microgravity affects astronauts’ health in adverse ways. However, less is known about the extent to which fibroblast differentiation during the wound healing process is affected by the lack of gravity. One of the key steps of this process is the differentiation of fibroblasts into myofibroblasts, which contribute functionally through extracellular matrix production and remodeling. In this work, we utilized collagen-based three-dimensional (3D) matrices to mimic interstitial tissue and studied fibroblast differentiation under simulated microgravity (sµG). Our results demonstrated that alpha-smooth muscle actin (αSMA) expression and translocation of Smad2/3 into the cell nucleus were reduced upon exposure to sµG compared to the 1*g* control, which suggests the impairment of fibroblast differentiation under sµG. Moreover, matrix remodeling and production were decreased under sµG, which is in line with the impaired fibroblast differentiation. We further investigated changes on a transcriptomic level using RNA sequencing. The results demonstrated that sµG has less effect on fibroblast transcriptomes, while sµG triggers changes in the transcriptome of myofibroblasts. Several genes and biological pathways found through transcriptome analysis have previously been reported to impair fibroblast differentiation. Overall, our data indicated that fibroblast differentiation, as well as matrix production and remodeling, are impaired in 3D culture under sµG conditions.

## 1. Introduction

The current interest in spaceflight and long-term stays in space is increasing. However, little is known about how the lack of gravity will affect the health of space travelers. It has been shown that spaceflight and microgravity conditions can cause adverse effects on the immune system, neurological function, and bone density [1,2,3]. Several in vitro and animal studies report problems such as skin irritation and impaired wound healing under microgravity conditions [4,5,6,7,8,9], suggesting that this process could also be altered in astronauts during spaceflight. 

Wound healing is a dynamic process involving many cell types (e.g., immune cells, keratinocytes, epithelial cells and endothelial cells), each with distinct roles in the regulation of different wound healing phases, namely homeostasis, inflammation, proliferation and remodeling [10]. Throughout the process, any alterations of multiple exogenous factors, including the extracellular matrix (ECM) composition and the spatio-temporal presence of soluble mediators in the wound and its periphery, can negatively influence the mechanisms of tissue repair. Dysregulation of these mechanisms can lead to improper or impaired wound repair [11].

Fibroblasts are the key players in wound healing during the tissue repair process [12,13]. During this process, fibroblasts differentiate into myofibroblasts in the presence of transforming growth factor beta 1 (TGF-β1) [14]. TGF-β1 is secreted by a multitude of cells when a tissue is injured. Upon TGF-β1 activation, the TGF-β1 pathway causes Smad2/3 proteins to translocate to the nucleus and initiate the myofibroblast differentiation program [15,16,17]. Myofibroblasts are characterized by high expression of alpha smooth muscle actin (αSMA), which allows them to gain their contractile properties, thereby possessing the ability to physically remodel the ECM [18,19]. In addition, myofibroblasts are known to secrete a variety of extracellular matrix components, e.g., collagen, fibronectin, hyaluronic acid, as well as metalloproteinases, all facilitating the tissue remodeling process [15,20,21].

Under real microgravity conditions, human fibroblast samples cultured during space flight reveal increased levels of collagen synthesis compared to control samples [22], while another report demonstrated a change in genes related cell stress signaling which could impact cell apoptosis and senescence [23]. The results from both studies are contradictory, as senescent fibroblasts are not able to produce high amounts of ECM components, but enhance their ECM catabolism [24]. To reproduce lack of gravity on Earth, simulated microgravity (sμG) platforms e.g., 2D and 3D clinostats [25], a rotating wall vessel (RWV) [26], and random positioning machines (RPM) [27] have been established [28]. Under sμG conditions, fibroblasts were found to exhibit changes in their cytoskeleton, ECM production (e.g., laminin and fibronectin), as well as altered growth behavior [5,29]. Other work also demonstrated a decrease in gene expression of αSMA and E-cadherin in primary human fibroblasts upon cultivation under sµG using a rotary cell culture system [30]. In addition, sμG diminished the expression of fibroblast cell adhesion proteins and cell alignment when these cells were cultured with different topographical cues [31]. Along with that, gene expression analysis of human fibroblasts exposed to sμG using a 3D-clinostat showed downregulation of cell cycle and proliferation genes such as ERB-B2 and p21^Cip1/Waf1^ [32]. In addition, RNA sequencing of fibroblasts exposed to sμG using 3D clinostat and radiation revealed the induction of genes related to genomic instability of the cell cycle [33]. Similar conditions of radiation and sμG exposure found decreased levels of apoptosis in fetal mouse skin [34]. These data indicate the impact of the lack of gravity on fibroblast behavior and functions. However, there is limited knowledge regarding fibroblast differentiation, subsequent ECM remodeling and production, and the molecular mechanisms involved in the tissue repair process under microgravity conditions.

In this work, studies whether sμG conditions affects fibroblast differentiation into myofibroblasts when compared to 1*g* (i.e., not loaded on the RPM, where g is Earth’s gravity at ground level). To ensure the physiological relevance of our model, we utilized a three-dimensional (3D) cell culture system based on a collagen matrix as a biomimetic tissue model. Three-dimensional collagen matrices allow us a more in depth understanding of mechanisms due to their structural complexity, and are widely used due to their ability to better mimic interstitial tissue compared to traditional 2D cell culture surfaces [35,36]. Currently, the effects of sμG on fibroblast differentiation and function in 3D culture has yet to be studied. Overall, our work provides a more physiologically relevant model into tissue repair mechanisms, especially on ECM remodeling, under sµG conditions.

## 2. Results and Discussion

**Figure 1:**
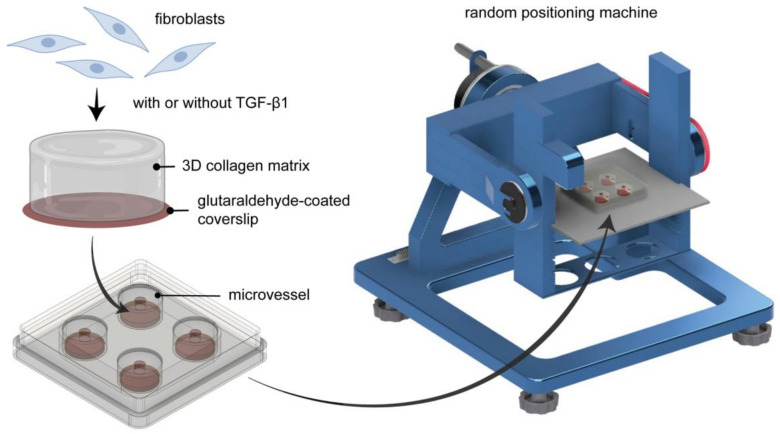
Schematic illustration of experimental setup. Fibroblasts were cultured in 3D collagen matrices and placed inside engineered biocompatible microvessels before being cultured either on 1*g* or on the random positioning machine (RPM). RPM is placed in a conventional cell culture incubator.

Fibroblast differentiation is a critical step during the tissue repair process [12,13], and microgravity has been reported to reduce the ability of fibroblasts to differentiate into myofibroblasts in 2D culture [29]. However, 2D culture poorly captures any features of the 3D microenvironments of the native tissue. In this work, we aimed to demonstrate the extent to which sμG affects the tissue repair process, particularly focusing on the differentiation of fibroblasts into myofibroblasts, by utilizing 3D collagen matrices as a biomimetic tissue model. Primary human dermal fibroblasts were cultured within collagen matrices placed inside an engineered cell culture microvessel [37], then conditioned using sμG. Controls were samples placed in microvessels but not conditioned with sµG (1*g*). As mentioned, the essential step in the tissue repair process is the differentiation of fibroblasts into myofibroblasts. To induce myofibroblast differentiation, cell culture medium was supplemented with 10 ng/mL of TGF-β1. After 3 days of cultivation, cells were analyzed in terms of differentiation state by means of αSMA expression, nuclear translocation of Smad2/3, transcriptome analysis using RNA sequencing (RNA-seq), matrix remodeling using a custom-made image analysis toolbox and cytokine secretion profile using multiplex bead-based ELISA. A schematic illustration of the experimental setup is depicted in Figure 1.

### 2.1. SµG Impaired Fibroblast Differentiation

**Figure 2:**
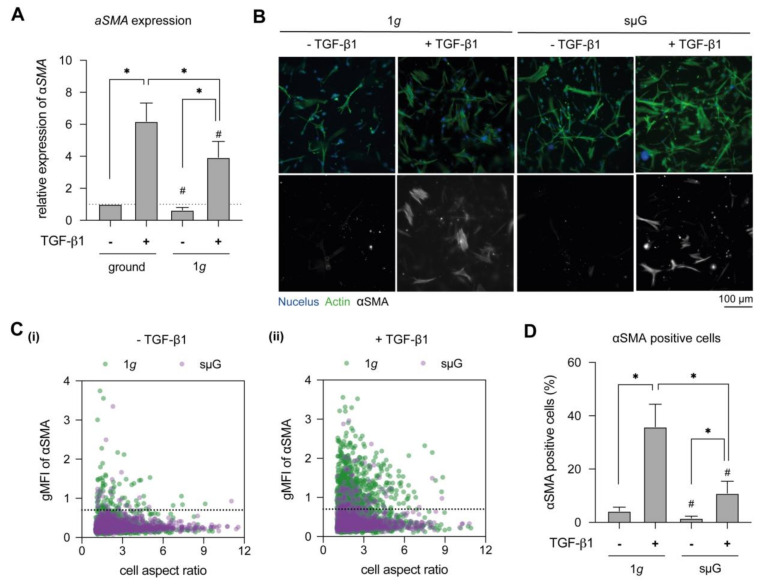
Myofibroblast differentiation in dependence of TGF-β1 stimulation under 1*g* and sµG. Fibroblasts were cultured for 3 days in the presence and absence of TGF-β1 under 1*g* and sμG conditions. (**A**) Cells were analyzed regarding αSMA expression using RT-qPCR. Gene expression analysis was performed in four replicates. (**B**) Representative image of fibroblasts stained with DAPI (blue), Phalloidin (green) and αSMA (grey) antibodies. (**C**) Quantitative analysis of geometric mean of fluorescence intensity (gMFI) of αSMA and cell aspect ratio using a custom-made image analysis toolbox. The dashed line in C(i) and C(ii) represents the cut-off value, below which are the majority of low αSMA expressing cells without TGF-β1 under 1*g* condition. (**D**) Percentage of αSMA positive cells. The image analysis was performed at least in triplicate with four positions per sample. Data are shown as mean +/− SD. * indicates significant *p* ≤ 0.005. The character # represents the significance level of *p* ≤ 0.05 when compared to 1g at similar condition.

To elucidate the effect of microgravity on fibroblast differentiation, we first studied *αSMA* gene expression, a prominent marker of myofibroblasts [15,38], using real-time quantitative polymerase chain reaction (RT-qPCR) (Figure 2A). As expected, we found significantly higher αSMA expression upon TGF-β1 stimulation for both 1*g* and sμG conditions, but a significant reduction in αSMA expression was observed under sμG conditions when compared to 1*g* conditions, whether sμG samples were TGF-β1 stimulated or not. To verify the gene expression results, cells were stained with αSMA antibodies and visualized using epi-fluorescence microscopy (Figure 2B). It was observed that fibroblasts expressed αSMA upon stimulation with TGF-β1 in both 1*g* and sμG conditions. By plotting mean fluorescent intensity of αSMA and cell aspect ratio (Figure 2C(i),(ii)), we saw a reduction in cell aspect ratio of fibroblast with TGF-β1 stimulation when compared to the unstimulated counterpart. This morphological observation is in line with other works [21,38,39], whereby undifferentiated cells were elongated, i.e., higher aspect ratio. By comparing αSMA-positive cells (Figure 2C(i),D), we found that few cells expressed αSMA in unstimulated conditions, which might be caused by heterogeneity of the fibroblast population. Comparably, the amount of αSMA-positive cells in unstimulated conditions remained relatively low when cultured under sμG conditions. Similarly, we found lower αSMA-positive cells upon TGF-β1 stimulation under sμG conditions when compared to 1*g* conditions (Figure 2C(ii),D). Our results corroborate well with a report that demonstrated the reduction of αSMA expression in 2D culture under sµG conditions [30]. However, the mechanism remains unclear. We hypothesize that a lower activation of the TGF-β1/Smad pathway might be involved in the limited fibroblast differentiation under sμG conditions.

### 2.2. Nucleus Translocation of Smad2/3 Was Reduced in sµG Conditions

**Figure 3:**
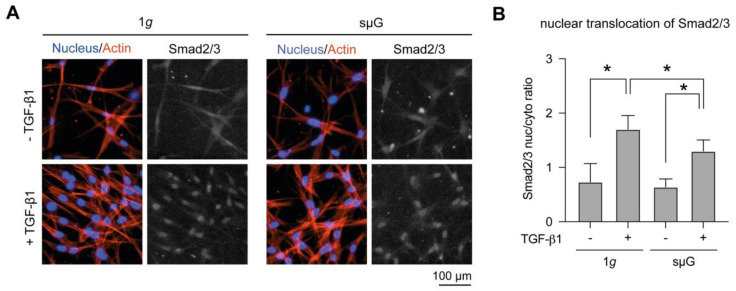
Nuclear translocation of Smad2/3 in dependence of TGF-β1 stimulation under 1*g* and sµG. (**A**) Representative image of cells stained with Hoechst-33342 (blue), Phalloidin (red) and Smad2/3 (grey) antibodies. (**B**) The quantitative image analysis was performed at least in triplicate with four positions per sample. Data are shown as mean +/− SD. * indicates significant *p* ≤ 0.005.

Upon TGF-β1 binding to its receptors, Smad2/3 is phosphorylated, binds to Smad4 and the complex is translocated from the cytoplasm into the nucleus [15]. A positive correlation of αSMA expression and nuclear translocation of Smad2/3 has been reported in human dermal fibroblasts [17,40]. To address whether the impairment of fibroblast differentiation in sµG conditions is caused by a reduction in Smad2/3 signaling, we analyzed nuclear translocation of Smad2/3 using immunocytostaining and quantitative image analysis. As shown in Figure 3A, Smad2/3 was located in the cytoplasm and less in the nucleus in fibroblasts without TGF-β1 stimulation in both 1*g* and sμG conditions. After TGF-β1 stimulation, Smad2/3 was translocated into cell nuclei in both conditions, suggesting the activation of TGF-β1/Smad pathway. By quantitative analysis of the obtained images, we found a significant increase of Smad2/3 translocation upon TGF-β1 stimulation in both conditions (Figure 3B). However, a significant reduction of Smad2/3 nuclear translocation was observed when compared to fibroblasts treated with TGF-β1 in sµG conditions. Our results suggest that the reduction of Smad2/3 activation might be involved in fibroblast differentiation.

### 2.3. ECM Remodelling and Production Are Reduced in sµG Conditions

**Figure 4:**
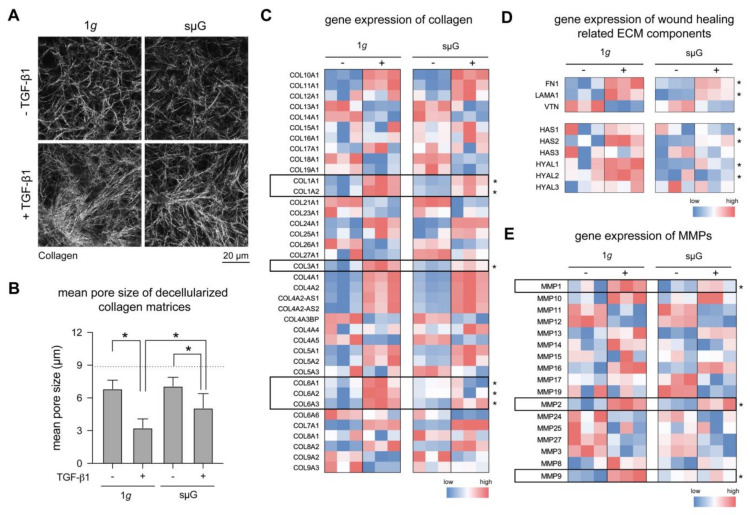
Matrix remodeling and ECM gene expression under 1*g* and sµG conditions. (**A**) Representative images of decellularized collagen matrices. Matrices were analyzed regarding (**B**) pore size. The image analysis was performed at least in triplicate with four positions per sample. Data are shown as mean +/− SD. * indicates significant *p* ≤ 0.05. Gene expression using RNA-Seq data of (**C**) collagens, (**D**) wound healing related ECM factors, and (E) matrix metalloproteinases (MMPs) in both 1*g* and sμG conditions. Data are shown as a heatmap using colors on a scale from red (high expression) to blue (low expression). Statistical significance test was performed for TGF-β1-treated samples between 1*g* and sµG conditions for Figure 4C–E, which is indicated by * for *p*-value ≤ 0.05. The experiment was performed in three replicates.

A key feature of myofibroblasts is their ability to remodel the ECM and secrete new ECM components to facilitate tissue repair [21,41]. To investigate the effects of sμG on this process, we first studied matrix remodeling of fibroblasts and myofibroblasts. After 3 days of cell cultivation, collagen matrices were decellularized, stained with 5-(and-6)-Carboxytetramethylrhodamine succinimidyl ester (TAMRA-SE) and visualized using confocal microscopy (Figure 4A). Matrix porosity, as characterized by mean pore size, was calculated using a custom-built image analysis software. We found no significant changes in matrix porosity in fibroblast matrices on both 1*g* and sµG conditions (Figure 4B). In contrast, myofibroblast matrices in sµG had a significantly larger pore size when compared to 1*g* conditions. This suggests that the ability of myofibroblast to remodel the collagen matrix is reduced under sµG conditions. This result is in line with the reduction of αSMA expression in sµG, as demonstrated in Figure 2.

We next analyzed the production of collagen and wound healing related ECM components, as well as matrix metalloproteinases (MMPs) using RNA-Seq data. Upon differentiation, fibroblasts produce high amounts of matrix components, especially collagen, fibronectin and hyaluronic acid [21,40,42]. As shown in Figure 4C, major collagen types known to be expressed higher during tissue repair, namely *Coll1a1, Coll1a2, Coll3a1, Coll6a1, Coll6a2* and *Coll6a3*, were found to have increased expression upon fibroblast differentiation when compared to undifferentiated fibroblasts, but this increased expression was reduced in sµG. In addition, we found a similar trend to collagen expression in other ECM components, namely *FN1* (fibronectin), *LAMA1* (laminin), *VTN* (vitronectin), as well as *HAS1-3* and *HYAL1-3* (hyaluronic acid synthesis and degradation enzyme), as shown in Figure 4D. Our data contradicts other work which reported that human fibroblasts cultured on 2D substrate demonstrated higher expression of laminin and fibronectin after sµG exposure [5,29]. It was hypothesized that the increase of fibronectin could be the cause for impaired ECM rebuilding [29]; however, it has been shown in a 3D cell culture model that fibronectin can enhance ECM remodeling and fibroblast migration [19]. The discrepancy between our results and other works might be because of cell culture dimensionality, biophysical and biochemical properties of the cell culture substrate, and cell culture conditions. Besides the production of ECM components, MMPs are important enzymes during the ECM remodeling process. We found major MMPs involved in this process, namely *MMP1*, *MMP2* and *MMP9*, to be less expressed in fibroblasts when compared to myofibroblasts in both sµG and 1*g* conditions (Figure 4E). Again, we observed a reduction in gene expression of these MMPs in myofibroblasts in sµG when compared to the 1*g* counterpart.

Overall, we found that matrix remodeling through the expression of collagen and other ECM components, as well as matrix metalloproteinases (MMPs), were reduced in myofibroblasts under sµG conditions when compared to cell culture at 1*g*. These results support the impairment of fibroblast differentiation under sµG, as demonstrated in the previous sections. This emphasizes the effects of sµG conditions on fibroblast differentiation and ECM remodeling in our biomimetic cell culture model. 

### 2.4. RNA-Seq Revealed Minimal Change in Transcriptomic of Fibroblast under 1g and sµG Conditions

As demonstrated above, fibroblast differentiation and function are impaired in sµG. Using RNA-Seq we analyzed the transcriptome of fibroblasts and myofibroblasts, both under 1*g* and under sµG, in an attempt to explain the impairment in fibroblast differentiation under sµG at a transcriptomic level.

**Figure 5:**
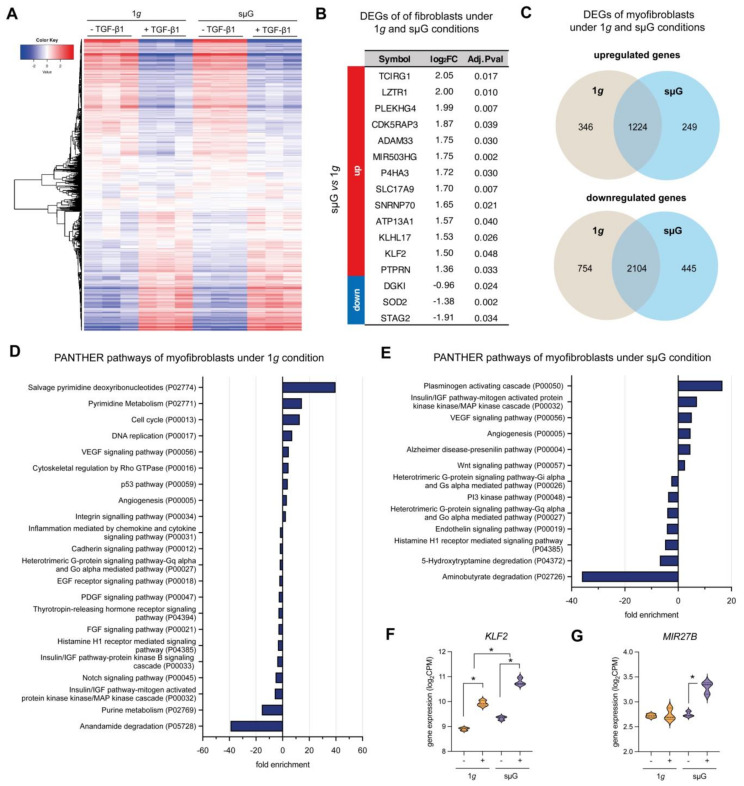
RNA-Sequencing analysis of fibroblast and myofibroblast under 1*g* and sµG conditions. (**A**) Heat map of overall gene expression levels in fibroblasts and myofibroblasts under 1*g* and under sµG. DEGs were analyzed using DESeq2 with FDR cutoff ≤ 0.05 and FC ≥ 2.0 using DESeq2 (**B**) Table of differentially expressed genes (DEGs) with log_2_ fold change (log_2_FC) and adjusted P-value of fibroblasts cultured under 1*g* and under sµG. Up- and downregulated genes were compared between sµG and 1*g* conditions. (**C**) Venn diagram of up- and down-regulated DEGs of myofibroblast cultured under 1*g* and under sµG. The DEGs were analyzed for enriched biological pathways using statistical overrepresentation tests using the PANTHER pathway for (**D**) 1*g* and (**E**) sµG conditions. Gene expression of specific DEGs which has been reported to be involved in inhibition of fibroblast differentiation, namely (**F**) *KLF2* and (**G**) *MIR27B.* Data are shown as a violin plot; * significance level of *p* < 0.05. The experiment was performed with three replicates.

As shown by the heat map in Figure 5A, minimal changes in transcriptome levels between 1*g* and sµG conditions in both fibroblasts and myofibroblasts were found. By analyzing differentially expressed genes (DEGs) in fibroblasts between both cell culture conditions, we found only 16 DEGs with a fold change (FC) higher or equal to two, and a false discovery rate (FDR) smaller or equal to 0.05 (Figure 5B). Only three genes, namely *DGKI*, *SOD2* and *STAG2* were reduced in sµG condition when compared to the 1*g* condition. We could not assign DEGs of fibroblasts in both cell culture conditions to any specific biological pathways. It should be noted that our RNA-Seq experiment was limited by the number of samples (*n* = 3) and therefore this could affect the poor performance of the DEGs analysis. However, our finding is consistent with another report demonstrating that few genes (82 genes related to oxidative stress, DNA repair and fatty acid oxidation) were differentially expressed in WI-38 human fibroblasts cell line after 5 days of spaceflight [23] taking into account the effects of radiation as well. Other work by Zhang et al. also reported minimal changes in the transcriptome of human fibroblasts upon spaceflight [43].

Upon fibroblast differentiation into myofibroblasts using TGF-β1, we found 346 and 249 DEGs upregulated under 1*g* and sµG respectively, while cells in both conditions shared 1224 upregulated genes (Figure 5C). In contrast, there were 445 and 754 downregulated DEGs under 1*g* and sµG, respectively, with 2104 downregulated DEGs shared by both conditions (Figure 5C).

We further assigned the up- and downregulated genes of myofibroblasts into biological pathways by a statistical enrichment test using the PANTHER database [44]. In Figure 5D, pathways related to up- and downregulated DEGs of fibroblast under 1*g* conditions are depicted. Biological pathways, such as salvage pyrimidine deoxyribonucleotide, pyrimidine metabolism, cell cycle and DNA replication, were upregulated. This corroborates well with the observation that myofibroblasts exhibit enhanced proliferative capability [21,45]. Under sµG conditions, the plasminogen-activating cascade, the insulin/IGF pathway-MAPKK-MAPK cascade, the VEGF signaling pathway and angiogenesis were upregulated, while aminobutyrate degradation, 5-hydroxytryptamine degradation and histamine H1-mediated signaling pathways were downregulated (Figure 5E). However, the direct link between these biological pathways and fibroblast differentiation and behavior are unexplored.

Using RNA-Seq we reveal several possible pathways which might be involved in this process. The plasminogen activation cascade was found highly upregulated under sµG conditions, but not under 1*g*. It has been reported that the plasminogen activation cascade could regulate fibroblast apoptosis and there may be a potential role of TGF-β1/PAI-1 in promoting (myo)fibroblast survival in chronic fibrotic disorders [46]. Another pathway that might be involved in the impairment is the Insulin/IGF pathway-MAPKK-MAPK cascade, which was found to be upregulated under sµG conditions but downregulated under the 1*g* condition. IGF signaling is known for selective suppression of Smad3 activation [47]. In addition to both pathways, we found two specific DEGs which were reported to inhibit TGF-β1 signaling, namely *KLF2* (Figure 5F) and *miR-27b* (Figure 5G). *KLF2* was found to have higher expression under sµG when compared to 1*g* conditions. *KLF2* is known to exert a negative feedback on TGF-β1/Smad signaling [48] and has been shown to suppress TGF-β1 mediated signaling [49,50]. On the other hand, miR27b has been identified as the target for TGF-β receptor 1 and Smad2, which, in turn, results in inhibition of fibroblast differentiation [51]. Although the aforementioned evidence suggests impairment of fibroblast differentiation, statistical overrepresentation tests did not provide biological pathways specifically involved in the impairment of fibroblast differentiation under the sµG condition. We also further acknowledge that the impairment of fibroblast differentiation might be due to reduced physical interactions of proteins in the suG condition, which might inhibit the incorporation of αSMA into actin fibrils, as also seen in the suppression of the fibril formation in other proteins e.g., amyloid [52,53]. This requires further extensive investigation.

## 3. Conclusions

Tissue repair and wound healing are adversely impacted by space travel and exposure to microgravity. Our data shows that fibroblast differentiation, a key process of wound healing, is impaired in 3D cell culture upon exposure to sµG. Differentiation of fibroblasts into myofibroblasts using TGF-β1 showed less αSMA expression and reduced nuclear translocation of Smad2/3 in cells differentiated in sµG relative to 1*g* controls. In addition, RNA-Seq analysis did not reveal a large number of changes between fibroblasts differentiated under 1*g* or sµG conditions but showed a general decrease in expression of collagen and ECM factors in differentiated fibroblasts under the sµG condition relative to 1*g* controls. While specific pathways were shown to be downregulated under sµG conditions, further investigations are needed to elucidate the exact pathways and genes that are involved in the impairment of the wound healing process in microgravity conditions. 

## 4. Materials and Methods

### 4.1. Reconstruction of 3D Collagen Matrices

Three-dimensional collagen matrices for cell culture of fibroblasts were made by mixing rat tail type I collagen (Advanced BioMatrix, Inc. San Diego, CA, USA), 500 mM phosphate buffer (Sigma-Aldrich, St. Louis, MO, USA) and 0.1% acetic acid (Sigma-Aldrich, St. Louis, MO, USA) as a collagen solution at a concentration of 2 mg/mL, as previously published [54]. The prepared collagen solution was placed onto a glutaraldehyde-coated coverslip (13 mm in diameter; VWR, Darmstadt, Germany), allowing covalent binding of the collagen matrix via lysine side chain [55]. Collagen fibrillogenesis occurred by placing the coverslips at 37 °C, 5% CO_2_ and 95% humidity. Afterwards, the 3D collagen matrices were washed three times with phosphate buffer saline (PBS; Thermo Fisher Scientific Inc, Leicestershire, UK) and kept in PBS prior to use.

### 4.2. Cell Culture of Fibroblasts and Myofibroblast Differentiation

Primary human dermal fibroblasts were maintained in Dulbecco’s Modified Eagle Medium (DMEM) cell culture medium supplemented with 10% fetal bovine serum (FBS) and 1% penicillin/streptomycin at 37 °C, 5% CO_2_ and 95% humidity (standard cell culture conditions). Cell culture medium and supplements were all purchased from Gibco, Invitrogen, Thermo Fisher Scientific Inc, Dreieich, Germany.

For all experiments, the 3D collagen matrices were placed into four well-plates (Thermo Fisher Scientific Inc, Dreieich, Germany). 1 × 10^5^ fibroblast cells were seeded onto 3D collagen matrices and kept at standard cell culture conditions for 2 h to allow for cell attachment. Afterwards, the cell culture medium was removed and biocompatible microvessels were placed onto the well plate, as previously described [37]. Subsequently, fresh cell culture medium was added into the microvessel. For fibroblast differentiation, cell culture medium was supplemented with 10 ng/mL TGF- β1(Biolegend, San Diego, CA, USA) [21]. Cells were then cultured for 3 days at 1*g* or sµG conditions in an incubator at standard cell culture conditions.

### 4.3. Setting up of Random Positioning Machine

All experiments using sµG were performed on desktop Random Positioning Machine (RPM) (Airbus Defence and Space Netherlands B.V., Leiden, The Netherlands). The RPM was operated in 3D random mode, using random motion and random direction, maintaining an average velocity of 60 deg/s. Four-well cell culture well plates were placed at the center of the rotation, as previously described [37]. All experiments using the RPM were performed in an incubator under standard cell culture conditions.

### 4.4. Cell Staining and Qualitative Image Analysis 

For cell staining, microvessels were removed from the wells and cells were fixed with 4% paraformaldehyde (Biolegend, San Diego, CA, USA) for 10 min and subsequently permeabilized with 0.1% Triton X100 (Merck KGaA, Darmstadt, Germany) for 10 min. Cells were washed three times with PBS after each step. Afterwards, cells were stained with Phalloidin conjugated with Alexa Fluor 594 (dilution 1:250 in PBS; Invitrogen, Carlsbad, CA, USA), and Hoechst-33342 (dilution 1:10,000 in PBS; Invitrogen, Carlsbad, CA, USA). 

For additional staining of αSMA, cells were blocked with 1% bovine serum albumin for 1 h at room temperature, incubated with mouse anti-human αSMA (dilution 1:250 in PBS; Biolegend, San Diego, CA, USA) overnight at 4 °C, and incubated with goat anti-mouse IgG conjugated with Alexa Fluor 488 (dilution 1:250 in PBS; Invitrogen, Waltham, MA, USA) for 2 h.

For additional staining of Smad2/3, cells were blocked with 1% bovine serum albumin for 1 h at room temperature, incubated with mouse anti-human Smad2/3 conjugated with Alexa Fluor 488 (dilution 1:250 in PBS; Santa Cruz Biotechnology Inc, Dallas, TX, USA) overnight at 4 °C.

Cell imaging was performed using a Lionheart FX automated microscope (BioTek, Winooski, VT, USA) using a 10× objective. Stacked images were gathered at a z-interval of 5 µm with overall z-layer of 500 µm. The representative images are of cells at the z-layer approximately 10–20 μm below the collagen surface, where many cells are located. Quantitative image analysis was done using an automated custom-built 3D cell analysis software [56]. First, single cells were masked using Phalloidin fluorescence. Cell position in 3D space was defined by the maximal fluorescence intensity of the Hoechst-33342 across the z-layer. Afterwards, the geometric mean of fluorescence intensity (gMFI) of αSMA and Smad2/3 was quantified for each cell. For nuclear translocation of Smad2/3, the ratio of gMFI of Smad2/3 within the nucleus (Hoechst-33342 area) and cell cytoplasm (Phalloidin area) was calculated. For both αSMA and Smad2/3 quantification, image analysis was performed at least in triplicate with four positions per sample. 

### 4.5. Quantitative Analysis of Matrix Remodeling

Collagen matrices were decellularized by osmotic shock through incubation with distilled water for 1 h, as previously published [57]. Afterwards, matrices were stained with 50 µM of 5-(and-6)-Carboxytetramethylrhodamine succinimidyl ester (TAMRA-SE, Sigma-Aldrich, Schnelldorf, Germany) and visualized by confocal laser scanning microscope (cLSM) (SP8; Leica, Wetzlar, Germany) using 40× water immersion objective (Leica, Wetzlar, Germany), as published elsewhere [55]. The cLSM stacked images were gathered with a z-interval of 5 µm throughout the matrices. For the quantification of pore size of collagen matrices, stacked images of four different positions of each matrix condition were analyzed using a custom-built MATLAB script (MATLAB 2020a; MathWorks, USA) (publicly accessible at https://git.sc.uni-leipzig.de/pe695hoje/topology-analysis (accessed on: 15 September 2021)) [55]. Experiments were performed in triplicate.

### 4.6. RNA Isolation and Gene Expression Analysis

Gene expression analysis was performed using an established protocol, as published [21]. Briefly, RNA was extracted using TRIzol (Invitrogen, Thermo Fisher Scientific, Inc., Dreieich, Germany), followed by chloroform extraction (Sigma-Aldrich, Schnelldorf, Germany) using the manufacturer’s protocol. The RNA concentration and purity (the ratio of absorbance at 260 nm and 280 nm) were quantified using Nanodrop (Thermo Fisher Scientific, Inc., Dreieich, Germany). RNA was subsequently converted into complementary DNA (cDNA) using a high-capacity cDNA reverse transcription kit (Applied Biosystems, Thermo Fisher Scientific, Inc., Dreieich, Germany). The primers used in this study were synthesized from Bioneer Inc. (Daejeon, South Korea) qPCR was performed using the SYBR Green PCR Master Mix (Applied Biosystems, Thermo Fisher Scientific, Inc., Dreieich, Germany). The primer sequences used are listed in Table 1. The qPCR procedure was set as follows: denaturation for 5 min at 95 °C; 45 cycles of denaturation (95 °C, 15 s), annealing under primer-specific conditions (30 s), and target gene-specific extension (30 s at 72 °C). Fluorescence signals were measured for 20 s at 72 °C. To confirm the specificity of the PCR products, a melting curve analysis was performed at the end of each run. The RPS26 gene was used as a reference gene. The relative expression levels were calculated using the 2^−ΔΔCT^ method. Experiments were performed with at least four independent replicates.

### 4.7. RNA Sequencing and Analysis 

RNA for sequencing was isolated as mentioned in the RNA isolation and gene expression analysis, followed by a purification step using the RNeasy mini kit (Qiagen, Hilden, Germany) as described by the manufacturer’s protocol. RNA quantity and quality were quantified using Nanodrop (Thermo Fisher Scientific, Inc., Dreieich, Germany) and Qi RNA kit (Thermo Fisher Scientific, Inc., Dreieich, Germany). Samples were prepared with NEB Ultra II RNA kit (New England Biolabs, Ipswich, MA, USA) as per protocol instructions using NEBNext Poly(A) mRNA Magnetic Isolation module (New England Biolabs, Ipswich, MA, USA), and uniquely dual indexed. The resulting libraries concentration, size distribution, and quality were assessed on a Qubit 4 fluorometer (Thermo Fisher Scientific, Inc., Dreieich, Germany) with a dsDNA high sensitivity kit (Invitrogen, Carlsbad, CA, USA) and on a 4200 TapeStation using a High Sensitivity D5000 kit (Agilent, Santa Clara, CA, USA). Based on these results, libraries were normalized according to their molarity and pooled, then quantified with a library quantification kit for Illumina platforms (Roche, Basel, Switzerland) on a StepOnePlus qPCR machine (Thermo Fisher Scientific, Inc., Dreieich, Germany). Finally, pooled libraries were loaded at 350pM with 1% PhiX on S2 FlowCell, and paired end sequenced (2 × 150 bp) on a NovaSeq 6000 next generation sequencer (Illumina, San Diego, CA, USA). RNA-Seq was performed in triplicate.

Raw FASTQ sequenced reads were first assessed for quality using FastQC v0.11.5 (available online at http://www.bioinformatics.babraham.ac.uk/projects/fastqc/ (accessed on: 15 September 2021)) [58]. The reads were then passed through Trimmomatic v0.36 [59] for quality trimming and adapter sequence removal with the parameters (*ILLUMINACLIP: trimmomatic_adapter.fa:2:30:10 TRAILING:3 LEADING:3 SLIDINGWINDOW:4:15 MINLEN:36*). The surviving trimmed read pairs were then processed with Fastp [60] in order to remove poly-G tails and Novaseq/Nextseq specific artefacts. Following the quality trimming, the reads were assessed again using FastQC. Post QC and QT, the reads were aligned to the human reference genome GRCh38.p4 using HISAT2 [61] with the default parameters, and additionally by providing the *–dta* flag. The resulting SAM alignments were then converted to BAM format and coordinate sorted using SAMtools v1.3.1 [62]. The sorted alignment files were then passed through HTSeq-count v0.6.1p1 [63] using the following options (*-s no -t exon -I gene_id*) for raw count generation. Concurrently, the sorted alignments were processed through Stringtie v1.3.0 [64] for transcriptome quantification. Briefly the process was: stringtie -> stringtie merge (to create a merged transcriptome GTF file of all the samples) -> stringtie (this time using the GTF generated by the previous merging step). Finally, Qualimap v2.2.2 [65] was used to generate RNA-Seq specific QC metrics per sample.

RNA-Seq data were merged using the NASQAR toolbox (publicly accessible at http://nasqar.abudhabi.nyu.edu/ (accessed on: 15 September 2021)) [66] and the analysis was performed using iDEP 0.93 (http://bioinformatics.sdstate.edu/idep93/ (accessed on: 15 September 2021); publicly accessible by South Dakota State University) [67]. For the analysis of differential expressed genes (DEGs), DEGs were analyzed with FDR cutoff ≤ 0.05 and FC ≥ 2.0 using DESeq2 [68]. The DEGs were analyzed for enriched biological pathways using statistical overrepresentation test (PANTHER pathways) by the online tool PANTHER 14.0 (publicly accessible at http://pantherdb.org (accessed on: 15 September 2021)) [44].

### 4.8. Data and Statistical Analysis 

Statistical significance was determined by two-way ANOVA followed by Tukey’s post hoc test using Prism 9 (GraphPad Software Inc., San Diego, CA, USA) and the level of significance was set to *p* ≤ 0.05. Unless otherwise stated, all experiments were performed with at least three replicates and data are represented as mean ± standard deviation (SD).

## Figures and Tables

**Table 1:** RT-qPCR primer sequence.

Genes	Forward Primer Sequence (5′ → 3′)	Reverse Primer Sequence (5′ → 3′)	Accession Number
*RPS26*	CAATGGTCGTGCCAAAAAG	TTCACATACAGCTTGGGAAGC	NM_001029
*αSMA (ACTA2)*	AGACCCTGTTCCAGCCATC	TGCTAGGGCCGTGATCTC	NM_001141945.1

## Data Availability

Data will be made available on request.
